# *Verbascum ponticum* (Stef.) Extract Induces Lung Cancer Apoptosis via Mitochondrial-Dependent Apoptosis Pathway

**DOI:** 10.3390/life14111520

**Published:** 2024-11-20

**Authors:** Pawan S. Faris

**Affiliations:** 1 Department of Biology, College of Science, Salahaddin University-Erbil, Erbil 44001, Kurdistan Region of Iraq, Iraq; faris.pawan@unipv.it; 2 Department of Biology, Cihan University-Erbil, Erbil 44001, Kurdistan Region of Iraq, Iraq; 3 Department of Brain and Behavioral Sciences, University of Pavia, 27100 Pavia, Italy

**Keywords:** natural product, lung cancer, mitochondrial dysfunction, AIF, proliferation, viability

## Abstract

Non-small-cell lung carcinoma remains a significant health concern due to its high incidence and mortality rates. Traditional medicines play a central role in cancer therapy, with plant-derived bioactive compounds being studied for their potential to offer fewer side effects than conventional treatments. In traditional Kurdish medicine, different *Verbascum* species are used to treat burns, inflammation, and other conditions. While some species extracts have shown cytotoxic effects against several cancer cell lines like A549, the efficacy and mechanisms of action of the other species like *Verbascum ponticum* (*V. ponticum*) remain to be elucidated. Therefore, this study aimed to explore the effect of *V. ponticum* (Stef.) extract, collected from the Kurdistan region of the Iraq mountains, on A549 cells. A comprehensive approach was employed, utilizing immunocytochemical and functional analyses to assess apoptotic morphology, DNA fragmentation, alongside assays for cellular and mitochondrial function, proliferation, and viability. Additionally, the study investigated AIF mitochondrial translocation and evaluated mitochondrial membrane potential using the Rhodamine 123 assay. The results showed that the *V. ponticum* flower extract induced mitochondrial-mediated apoptosis in A549 cells via disruption of mitochondrial membrane potential, release of AIF, and translocation to the nucleus, independently of the caspase-3-activation pathway. These findings emphasize the potential of *V. ponticum* in lung cancer strategic treatments, meriting further phytochemical studies to identify the bioactive compounds it contains.

## 1. Introduction

In 2020, overall lung cancer was reported as the second most common cause of cancer-related deaths worldwide, accounting for 2.21 million cases and 1.80 million fatalities (World Health Organization in 2020). Non-small-cell lung carcinoma (NSCLC), a primary type of lung cancer that comprises nearly 85% of the cases, remains a considerable global health concern due to its high incidence and mortality rate [1]. NSCLC is often diagnosed at an advanced (metastatic), non-localized stage, resulting in a 5-year survival rate of only 56% [2]. Most ineffective chemotherapies are associated with the invasiveness and metastasis of malignant cells following developed chemoresistance, along with highly cytotoxic side effects particularly targeting proliferating cell populations [3,4]. Therefore, there is an urgent need for an effective therapeutic strategy with fewer side effects.

Plant natural products have long been a source of inspiration for novel pharmaceutical agents, and herbs with a history of traditional medicinal use continue to be invaluable reservoirs of bioactive compounds with diverse pharmacological potential. Many natural compounds and their derivatives are sources for most anti-cancer agents for antitumor therapy, including vinblastine, paclitaxel, epipodophyllotoxins, and camptothecin [5,6,7,8]. These compounds have been applied directly or as a prodrug to enhance the effectiveness of existing tumor therapy. The role of herbal bioactive compounds has been evidenced in the modulation of mitochondrial function for cancer therapy [9]. This opens new prospects for treating conditions related to mitochondrial dysfunction, through promoting mitochondrial biogenesis, controlling the fusion and division of existing ones, eliminating damaged mitochondria, improving energy production, and maintaining homeostasis [9,10,11,12]. Among these, Taxol, which is isolated from *Taxus baccata*, is the most potent anti-tumor source of drug that is widely used in the clinical treatment of breast cancer, ovarian cancer, and some head and neck cancers, as well as lung cancer [11,13,14]. Not only this but different Verbascum species, a plant from the Scrophulariaceae family, have a rich history of ethnomedicinal usage and are revered for their therapeutic properties [15,16,17,18]. Several *Verbascum* (V.) species, including *V. calvum*, are used in Kurdish traditional medicine for burns and skin diseases. Research by HI. M.Amin and colleagues found that a methanol extract of *V. calvum* flowers exhibits relevant anti-proliferative effects in A549 lung cancer cells. This extract is rich in phenolic compounds and iridoid glycosides, supporting its traditional use in Kurdistan as an anti-inflammatory remedy for skin wounds and burns [16]. Mitochondria is one of the cellular vital organelles involved in cancer development and aggressiveness and plays a central regulatory role in mediating the interplay between cellular metabolism and the progression of neoplastic transformations [19] by facilitating cellular adaptation to unfavorable tumor microenvironments [20].

Mitochondrial dysfunction is the core mechanism by which cancer cells become resistant and aggressive [21,22,23]. Targeting mitochondrial-associated pathways has attracted much attention in the field of anti-cancer therapies due to their essential roles in growth, escape death, apoptosis, and progression [24]. One of the mechanisms is proposed to be changes in the outer mitochondrial membrane permeability that lead to the release of multiple apoptosis-related molecules, including cytochrome c and over 40 others, including apoptosis-inducing factor (AIF), a 57 kDa mitochondrial oxidoreductase, which translocate to the nucleus and trigger cell death. This process results in DNA fragmentation without the involvement of caspase activation. Thus, AIF is considered a potential target for chemo-radiotherapeutic interventions in various malignancies [25]. In lung cancer, altered mitochondrial homeostasis is a prominent feature of chemoresistance, and the tumor cells are characterized by the ability to escape from programmed cell death mechanisms, such as apoptosis, which represents a critical form of oncogene dependency [26,27]. AIF-regulated mitochondrial respiration drives the progression of lung cancer; thereby, recent evidence showed that genetic deletion of the AIF in the NSCLC mouse model boosts the survivor rate [28]. Accordingly, targeting this signaling pathway might provide a novel prospect in the era of lung cancer treatments.

Other Verbascum species traditionally used by Kurdish local people for the same purpose include *Verbascum ponticum* (Stef.) (*V. ponticum*). However, whether and how this plant affects cancer cells, in particular NSCLC, and the mechanism by which the herb exerts its actions are unclear. Here in this study, we aim to address this gap in the literature by investigating the cytotoxic effects of *V. ponticum* flower extract on A549 cells, a type of NSCLC cell line. By elucidating the mechanisms underlying its anti-cancer properties, we seek to contribute to a better understanding of the therapeutic potential of this traditional remedy for cancer treatment.

## 2. Materials and Methods

### 2.1. Plant Collection

The *V. ponticum* (Stef.) whole plant parts were collected in April 2023 during the flowering season, from Bnari bamo-Kalar, Garmian region, in the Kurdistan region of Iraq. A voucher specimen (code no. 7301) was deposited at the Herbarium of Salahaddin University-Erbil (ESUH). The plant was identified by Dr. Abdulla Shukr at Salahaddin University-Erbil. Fresh flowers were air-dried under shade at room temperature (20–25 °C) until a constant weight. After drying, the flowers were finely powdered using a laboratory grinding mill, to provide a homogeneous powder, which was stored in bottles at room temperature until used.

### 2.2. Flower Extraction

The powdered flowers (150 g) were soaked in petroleum ether (3 × 300 mL) in a flask with occasional shaking in an ultrasonic bath for 10 min; subsequently, the flowers were left in the same solvent at room temperature for 3 h. The mixture was filtered through a Whatman filter paper, and the solvent was removed with a rotavapor under reduced pressure to give an oily residue (A, 0.52 g). The defatted flowers were placed in a flask and extracted with methanol (3 × 300 mL) in an ultrasonic bath for 10 min and then at room temperature for 3 h under continuous stirring. The combined methanol (MeOH) extracts were filtered, and evaporation under reduced pressure afforded a crude residue (sample B) (9.12 g). These residues were stored at 4 °C under light protection.

### 2.3. Cell Culturing

Human NSCLC cell lines (A549 and NCI-H460 (ATCC No. CLL-185)) and normal human bronchial epithelial cells (NL-20) (as a control condition) are grown in culturing flasks for several days at 37 °C under a humidified atmosphere supplied by 5% CO_2_ and 95% O_2_ and changing the liquid growth medium (RPMI 1640 or DMEM (Euroclone, Milan, Italy)) supplemented with 10% fetal bovine serum (FBS) (Gibco, Oxford, UK) and 0.005% L-glutamine, penicillin, and streptomycin (Life Technologies, Milan, Italy)) whenever needed. Cells were tested and deemed free of Mycoplasma infection by the 265 Universal Mycoplasma Detection Kit ATCC (30–1012 K). When the culture reached confluency, the monolayer adherent cells were collected by trypsinization with 0.25% trypsin and 0.02% EDTA (Life Technologies Inc., Carlsbad, CA, USA) to de-attach the cells from the culturing flask. The culture medium was changed twice a week and cellular homogeneity was evaluated microscopically every 24 h. Cells were cryopreserved in 90% FBS and 10% dimethyl sulfoxide (DMSO) and stored in liquid nitrogen for further experiments. All experiments were carried out on cell lines passaged 5–15 times.

### 2.4. Cytotoxicity and IC_50_

The cytotoxic effect of the flower extract was assessed by MTS [3-(4,5-dimethylthiazol-2-yl)-5-(3-carboxymethoxyphenyl)-2-(4-sulfophenyl)-2H-tetrazolium] assay, which is based on the reduction in the MTS yellow tetrazolium compound by NADPH-dependent dehydrogenase enzymes in active cells to a purple formazan salt. Briefly, A549, NCI-H460, and NL-20 cells were seeded at a density of 5 × 10^5^ cells. When they reached confluency, they were detached by trypsin, centrifugated (at 1000 rpm for 10 min), resuspended in a growth medium (1 mL), and counted. After a desired dilution, cells were plated in 96-well microplates (CellStar, Sigma Aldrich, Milan, Italy) at a density of 2 × 10^3^ cells in 100 µL of growth medium/well. After 2 h incubation at 37 °C, the growth medium was replaced with 100 µL of FBS-free medium, and the microplate was incubated for 24 h. For the A549 cells, five different dilutions (1 mL for each) with concentrations equal to 500, 50, 5, 0.5, and 0.05 µg/mL were prepared by diluting a stock solution of sample B (50 mg/mL MeOH) with the FBS-free medium. The medium was replaced with a solution (100 µL) of increasing sample concentration. The microplate was then further incubated for 24 h; subsequently, the sample containing medium was replaced with fresh test medium (FBS free) (100 µL) and 20 µL of MTS tetrazolium reagent (CellTiter 96^®^—AQueous One Solution Cell Proliferation Assay, Promega, Madison, WI, USA). After 2 h of incubation, the absorbance was measured at 490 nm using a plate reader (Bio-Rad Model 550 Reader, Bio-Rad, Hercules, CA, USA). The IC_50_ was calculated accordingly to decide the optimum dosage of the drugs for further studies. The IC_50_ concentration of the flower extract was subsequently used to assess its safety profile against normal human NL-20 cells.

### 2.5. Proliferation and Viability Assay

After IC_50_ detection, the lowest effective concentration of sample B (50.14 μg/mL) was further used for the rest of the assessments to evaluate the underlying mechanism of action in NSCLC cell lines. Briefly, the cells were treated with the extract and incubated for a further 24, 48, or 72 h. The trypan blue exclusion method was used for these assessments; the trypan blue stock was prepared at 0.4% diluted with PBS to 0.2%. Cells were washed with PBS, collected by trypsinization, centrifuged for 10 min 1000 rpm, resuspended with 1 mL of PBS, and eventually incubated for 1 min with trypan blue stock 1:1 dilution. Viable and non-viable cells were counted and visualized with upright light microscopy under 20× objective magnification. The percentage of viability was calculated as the following formula: percentage viability = (number of viable cells/total number of cells) × 100, in which the total number counting of cells was considered for the proliferation assessments. The results were expressed as an average number of cells (mean ± SEM) under each condition, with and without the plant extract. At least five replicates were performed for each condition (non-treated cells and treated cells).

### 2.6. Nuclear and Cellular Morphology

Apoptotic cells were detected by staining the A549 cells with nuclear DAPI. The effect of the *V. ponticum* flower extract on apoptosis induction was evaluated, as described previously [29]. Cells at a density of 2 × 10^3^ were seeded on a 15 mm round cover glass (CELLTREAT scientific) in a 12-well plate. After incubation at 37 °C for 24 h, the cells were treated with 50.14 μg/mL of sample B and incubated for a further 24, 48, or 72 h. Then ice-cold paraformaldehyde (PFA) 4% was added to each well for fixation of cells and incubated for 20 min at 37 °C. After being rewashed with PBS, the DAPI solution (0.1% of final 500 nM final concentration) (Sigma Aldrich) was added to each well and incubated for 7 min; the cells then were washed and directly visualized under the fluorescence microscopy (Carl Zeiss, Oberkochen, Germany) at the excitation and emission wavelengths of 360 nm and 460 nm, respectively. For each condition, control, and treatment, approximately 100 cells were visualized from 4 rounds of independent experiments. Cells containing condensed and/or fragmented nuclei were considered apoptotic cells. For the whole-cell morphology observation, treated and non-treated cancer cells were examined by inverted light microscopy. Eventually, the percentage of apoptosis index was calculated by considering the ratio of several cells in which the nucleus exhibited an apoptotic phenotype by total cell count.

### 2.7. DNA Fragmentation Assessment

A549 cells (2 × 10^3^) treated with or without the IC_50_ concentration of a methanol extract from the *V. ponticum* flower for 24 h were used to assess DNA fragmentation using DNA gel electrophoresis assay. The DNA molecular weight-based ladder was used (Invitrogen, Carlsbad, CA, USA). Subsequently, the cells were washed with PBS and centrifuged to obtain the cell pellet, then re-suspended in 0.5 mL of lysis buffer and incubated for 30 min at 37 °C. The lysate was centrifuged. Proteinase K (0.5 mg/mL) was added to the supernatant followed by incubation at 56 °C overnight. The pellet was centrifuged and dried at room temperature and further treated with 100 µg/mL RNAase A for 1 h at 37 °C. The DNA fragment separation was performed by 1.5 percent agarose gel electrophoresis containing ethidium bromide, and the bands were visualized under a UV light scanner.

### 2.8. Immunocytochemical Staining

A549 cells were grown for 24 h on 15 mm glass coverslips at a density of 2 × 10^3^. Then, they were treated with 50.14 μg/mL of plant extract (sample B) for a further 24 h. The samples were later fixed with 4% PFA in PBS and permeabilized with 0.2% ice-cold TRITON X100 (Sigma Aldrich) in PBS. After rinsing with PBS, the cells were subsequently incubated in a blocking solution of 1% BSA in PBS. Then they were incubated with anti-AIF Antibody (PA5-48108) (ThermoFisher Scientific, Milan, Italy) using a dilution of 10 µg/mL for 3 h at room temperature. The samples were rinsed with PBS and thereafter incubated with the secondary anti-rabbit-FITC (Sigma-Aldrich) diluted 1:500 in the buffer for 1 h at room temperature. The samples were mounted on microscope slides with 10 μL of Vectashield Hard-Set mounting medium containing DAPI (Novus Biologicals, Milan, Italy), to stain and visualize the nuclei, and the images were captured with a confocal microscope (Leica, Wetzlar, Germany) with a 63× water immersion objective. AIF-conjugated cells were excited with 543 nm, and the fluorescence was collected with a 530 nm emission filter.

### 2.9. Mitochondrial Membrane Potential (ΔΨm) Measurement

Mitochondrial membrane potential (ΔΨm) was measured by the rhodamine 123 retention, as previously described [30]. Briefly, A549 cells were plated on 15 mm glass coverslips in a 12-well plate at a density of 2 × 10^3^ and incubated for 24 h. Subsequently, the cells were subjected to 50 µg/mL of the extract and incubated for a further 6, 12, and 24 h, to assess the time window in which the plant extract exerted its effect. Untreated cells were considered to be the negative control. The rhodamine 123 (Sigma Aldrich R8004) dye was applied to the cells at final concentration of 5 mg/mL^−1^ and incubated for 45 min at 37 °C. The cells were then evaluated using Carl Zeiss fluorescent Microscopy, Germany, 20X magnification, to check the fluorescent signals. The excitation and emission were 488 and 515–575 nm for rhodamine 123 fluorescence detection, respectively.

### 2.10. Statistics

This study comprises a comparison between two groups or conditions, non-treated and treated cells with a *V. ponticum* plant extract. For each condition, separate sets of experiments were conducted. All the data were collected from cells deriving from at least 4 independent experiments. The obtained data were analyzed and evaluated statistically by both Origin 8.0 and GraphPad Prism 8.0.1 software. For the immunofluorescence analysis, the ImageJ software V 1.54 was used. The data are presented as mean ± SEM, while the number of cells analyzed is indicated in the corresponding bar histograms. The total number of cells analyzed was at least ~100 cells for each treated and non-treated condition.

## 3. Results

### 3.1. Verbascum ponticum (V. ponticum) Flower Extract Induces Cytotoxicity in Human Non-Small Cell Lung Cancer (NSCLC) A549 Cell Line

**Figure 1:**
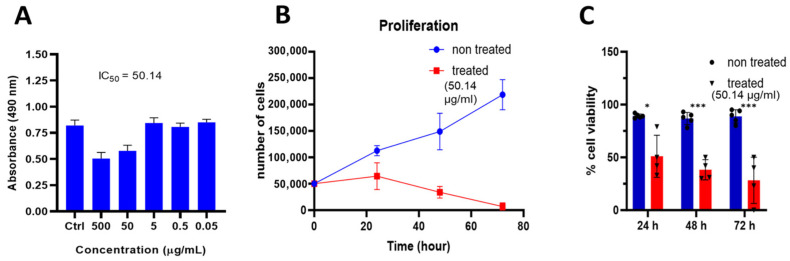
Effect of *Verbascum ponticum* (*V. ponticum*) flower extract on A549 cell proliferation and viability. (**A**) The histogram showing the IC_50_ value for the MTT cell viability assay. A549 cell viability decreased with increasing concentrations of *V. ponticum*-derived extracts. The effect of the extracts and formulation on 24 h was compared with non-treated cells (ctrl) or those treated with vehicle. (**B**) The line graph represents the proliferation capacity of A549 cells with and without 50.14 µg/mL of the herbal extract at 0, 24, 48, and 72 h incubation times. (**C**) The histogram represents the percentage of cell viability after 24, 48, and 72 h of incubation with and without 50.14 µg/mL of extract. The Student’s *t*-test was used for the statistical comparison. The data are expressed in mean ± SEM. (**B**,**C**) Blue color: non-treated cells (vehicle n = 5; red color: treated n = 4). Groups were considered statistically significant when * *p* ≤ 0.05 and *** *p* ≤ 0.001.

The growth inhibition percentages (the cytotoxic effect of the plant extract against A549 cells) of the tumor cells treated with the extract at different concentrations (500, 50, 5, 0.5, and 0.05 µg/mL) were calculated relative to the control (vehicle) (Figure 1A). The IC_50_ of sample B against the A549 cell line was 50.14 µg/mL, the minimum concentration that evoked anti-tumor activity. Subsequently, this concentration was utilized for further analysis, including testing the plant extract cytotoxicity on another human NSCLC cell line, NCI-H460 cells (Supplementary Figure S1), and cellular, functional, and molecular evaluation were assessed in the A549 cell line. The proliferation and viability of A549 cells significantly decreased after exposure to 50.14 µg/mL of sample B (flower for 24, 48, or 72 h), showing reduced cell counts and viability compared with the control group (Figure 1B,C). In the presence of extract the average count of tumor cells decreased notably to 64.250 ± 12,704, n = 4 at 24 h; 33.750 ± 5543, n = 4 at 48 h; and 6.875 ± 3442, n = 4 at 72 h, compared with the control condition (non-treated cells), in which the growth rate increased significantly to 112.50 ± 4787, n = 4 at 24 h; 148.750 ± 17,264, n = 4 at 48 h; and 218.50 ± 14,367, n = 4, at 72 h (Figure 1B). Furthermore, the percentage of cell viability exhibited a significant reduction following the administration of the herbal extract at all time points. The vital cells, appearing transparent or colorless, were significantly lower in percentage level than non-vital cells (blue), indicating a substantial decline in cell viability after the 24 h treatment, 85.6% in the control to 51% in cells treated with plant extract (Figure 1C). To further assess the safety profile of the flower extract, the human normal fibroblast, HFF-1 cells, were treated with 50.14 µg/mL of the flower extract for 24 h, and the results showed a lower cytotoxic effect as compared with the cancer 549 cell line (Supplementary Figure S1), while the non-significant difference was observed between non-treated and treated NL-20 cells (Supplementary Figure S1).

### 3.2. V. ponticum Flower Extract Influences A549 Cell Apoptosis

**Figure 2:**
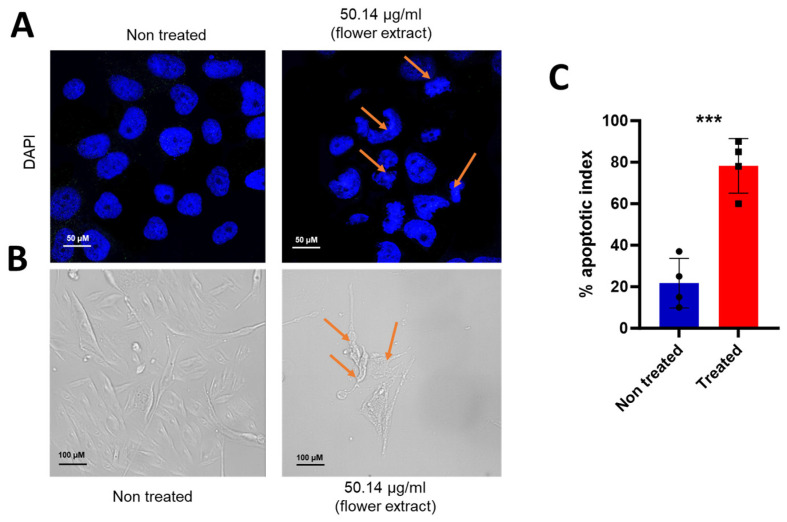
Effect of *V. ponticum* flower extract on the cell and nuclear apoptotic phenotype. (**A**) The photomicrographs depict the images of A549 cells with nuclear DAPI stain 24 h after treatment (plant extract at 50.14 µg/mL) or non-treated (vehicle). The arrows indicate the clear signs of nuclear condensation including the half-moon (crescent) shaped apoptotic nuclei. (**B**) Bright field light microscopy observation of cellular morphological alteration after 24 h of treatment. The cells treated with plants revealed clear signs of proapoptosis like blebbing of the cellular membrane and the typical apoptotic changes in the chromatin structure also with a clear reduction in the number of cells/visual field. The arrows indicate the apoptotic cells. (**C**) Graphical representation of the percentage of apoptotic indices. The apoptotic index for each test group was expressed as a percentage of the ratio of apoptotic cell number to the total cell number in 10 different fields. The Student’s *t*-test was used for the statistical comparison. Values are presented as mean ± SEM, n = 4, *** *p* ≤ 0.001.

To evaluate apoptotic markers, including chromatin condensation, remodeling, and nuclear fragmentation, nuclear DAPI staining was employed. A549 cells were subjected to treatment with the flower extract at a concentration of 50.14 µg/mL for a duration of 24 h. The findings depicted in Figure 2A unveiled marked morphological alterations in the nucleus, serving as a distinctive indicator of apoptosis in the treated cells compared with the control condition (non-treated cells). This discernible shift in nuclear morphology further corroborated the substantial cytotoxic effects of the extract on both cell proliferation and viability, as shown in Figure 1. Furthermore, the phase contrast microscopic analysis showed membrane blebbing, shrinkage, and floating cells post-treatment (Figure 2B). This observation highlights the multifaceted impact of the plant extract, not only on the quantitative aspects of cell numbers and viability but also on the qualitative aspects of cellular morphology. The apoptotic index calculation demonstrated a substantial increase in nuclear transformation in treated cells compared with the control, emphasizing the extract anti-proliferative effects on A549 cells as shown in Figure 2C (78.25% ± 6.56; 21.75% ± 5.96 for treated and non-treated A549 cells, respectively). In agreement with apoptosis, the results showed an apparent DNA breakage in A549 cells treated with *V. ponticum* compared with untreated cells (Supplementary Figure S2). These findings collectively highlight the potent apoptotic influence of *V. ponticum* flower extract on A549 lung cancer cells.

### 3.3. V. ponticum Flower Extract Induces Mitochondrial Dysfunction in A549 Cells by Promoting AIF Translocation to the Nucleus

**Figure 3:**
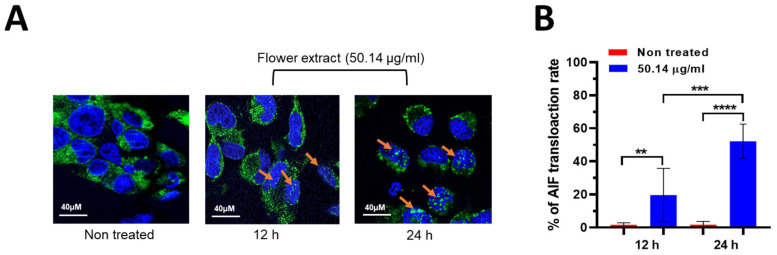
*V. ponticum* flower extract-induced nuclear translocation of apoptosis-inducing factor (AIF) in A549 lung cancer cells. The A549 cells were treated with the flower extract for 24 h. (**A**) The results shown are representative photographs of multiple experiments (n = 4, at least five coverslips observed/experiment). The nuclear translocation of AIF was demonstrated with immunofluorescence staining and laser scanning confocal microscopy—blue, DAPI-labeled nuclei; green, AIF. Orange arrows point to the nuclear AIF in the treated condition. (**B**) Summary of AIF translocation in the absence (non-treated or vehicle) and presence of the flower extract (50.14 µg/mL) incubation at two time points (12 and 24 h). Data were quantified by the number of cells positive for AIF green fluorescence among the total number of cells determined by DAPI staining. Data are shown as mean ± SEM; non-treated vs. treated and treated at 12 vs. 24 h time points were separately compared by Student’s *t*-test; n = 4, ** *p* ≤ 0.01, *** *p* ≤ 0.001, **** *p* ≤ 0.0001.

To unravel the molecular pathways influenced by the *V. ponticum* flower extract, AIF mitochondrial translocation to the nucleus was assessed after 12 and 24 h incubation with 50.14 µg/mL. The results illustrated in Figure 3A,B reveal a significant increase in the fluorescence intensity of AIF and colocalized into the nucleus of the treated cells compared to the control (non-treated). This compelling evidence suggests the translocation of AIF from the mitochondria to the nucleus following 12 h of exposure to the flower extract. Indeed, the percentage of AIF translocation rate was higher in treated cells (19.67 ± 6.098) compared to non-treated ones (1.701 ± 0.4137), the effect was significantly induced at 24 h incubation time point to 52.21 ± 3.906 (Figure 3B). AIF, a mitochondrial protein intricately involved in caspase-independent cell death, plays a crucial role in mitochondrial events during apoptosis. The alterations in AIF levels or their translocation from the mitochondria to the nucleus signify disruptions in mitochondrial function, contributing to cellular stress or dysfunction and eventually triggering the caspase-independent apoptosis pathway, respectively. To further confirm that apoptosis is caspase-independent, the detection of caspase-3 activity (known as an executioner caspase in apoptosis) revealed its inactivation, indicating that the apoptotic pathway was primarily independent of caspase-3 activation (Supplementary Figure S3). Staurosporine was used as a positive control to validate the assay, and its effectiveness in activating caspase-3 further supported the conclusion regarding the nature of apoptosis induced by *V. ponticum* extract [31].

### 3.4. Effect of the V. ponticum on Mitochondrial Membrane Potential (ΔΨm) in A549 Cells

**Figure 4:**
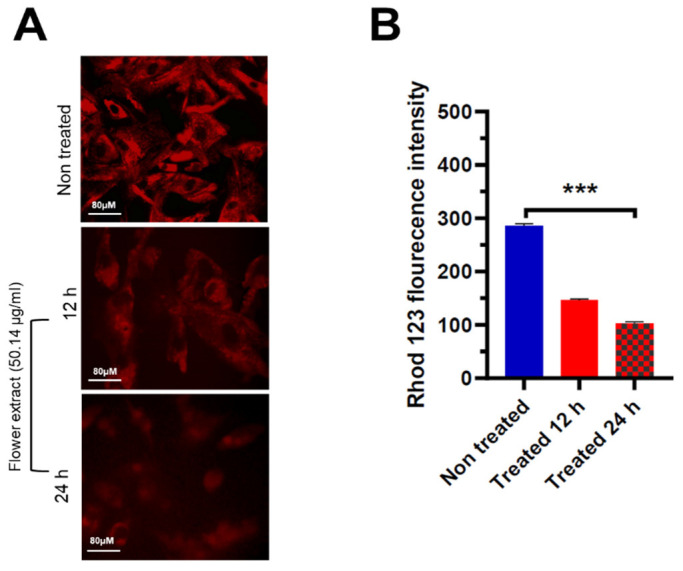
*V. ponticum* flower extract induces mitochondrial membrane depolarization in A549 cells. (**A**) Rhodamine 123 stained images depicting mitochondrial membrane potential (ΔΨm) of A549 cells treated with 50.14 μg/mL of plant extract for 12 and 24 h and non-treated (vehicle). (**B**) Histogram illustrating the quantification of rhodamine 123 fluorescence in all sets of A549 cells. n = 4. Values are presented as mean ± SEM analyzed by ANOVA, post hoc Dunnett’s, and *** *p* ≤ 0.001.

In the early apoptosis stage, the change of mitochondrial membrane potential (ΔΨm) is a marker for mitochondrial dysfunction. Accordingly, a rhodamine 123 assay was employed to evaluate ΔΨm in A549 cells to further investigate the induction of apoptosis by the herbal extract. The cationic probe, rhodamine 123, can be absorbed readily by vital cells and accumulated into the mitochondria. When the mitochondrial membrane is depolarized, it is considered an indication of early apoptotic events, which avoids any entry of the cationic probe into the mitochondria. According to this assay, the cells were exposed to rhodamine 123, and the fluorescence intensity was measured in the cells following the treatment (Figure 4A). Since this process was an early stage of apoptosis, the treated and control cells were evaluated starting from 12 h of incubation and, subsequently, the 24 h incubated cells. Almost a 50% decrease in ΔΨm with 50.14 μg/mL of the extract was observed, compared with the control group observed at 12 h incubation time, while at 24 h the fluorescence intensity decreased down to 23% in treated cells. The apoptotic index was notably higher in the treatment group at both 12 and 24 h compared with the negative control group (Figure 4B).

## 4. Discussion

Diverse medicinal plants showed convincing anti-cancer effects [16,32,33], mediated through different mechanisms. Molecular docking is utilized to understand the interactions and therapeutic properties of phytochemicals within target active sites [34]. Among these, targeting mitochondrial function is one of the potent targets in the field of cancer treatments [9,35]. The findings of this study unveil promising anti-cancer effects of *V. ponticum* flower extract against A549 cells. Notably, the plant was collected from the Kurdistan region mountains, where it has been traditionally used by local inhabitants for medicinal purposes, including skin burns and inflammation. Despite its long-standing traditional use, our study represents the first scientific investigation into its anti-cancer properties and mechanism of action against A549 cells.

The flower extract demonstrated significant inhibition of cell growth and proliferation in a dose-dependent manner, with an estimated IC_50_ of 50.14 µg/mL [36]. Treatment with the extract resulted in morphological alterations indicative of apoptosis, including chromatin condensation, remodeling, and nuclear fragmentation, accompanied by membrane blebbing and shrinkage observed through phase-contrast microscopy [35,37]. Additionally, the observed DNA fragmentation suggested that *V. ponticum* flower extract induced apoptosis. While DNA fragmentation is a hallmark of apoptosis, it can occur through either caspase-dependent or caspase-independent pathways. Supporting this, previous studies have demonstrated DNA fragmentation independently of caspase activity, such as with Okadaic acid in caspase-3 knockout cells, suggesting caspase-independent mechanisms [38]. Other substances, like granzyme B and Dioscin, have similarly triggered apoptosis via caspase-independent pathways by activating AIF translocation and inducing DNA fragmentation [39,40]. Given these findings, the apoptosis induced by *V. ponticum* extract may follow a similar pathway, potentially involving AIF. Additionally, studies showing that certain toxicants can induce apoptosis through caspase-independent pathways support the notion that *V. ponticum* may activate similar processes [41].

These findings underscore the extract’s multifaceted impact on cellular morphology and its ability to induce apoptotic pathways in the NSCLC cell line. Moreover, the study elucidates the mechanism underlying the apoptotic effects by revealing the translocation of AIF from mitochondria to the nucleus following treatment. This translocation indicates disruptions in mitochondrial function, ultimately triggering caspase-independent apoptosis pathways [42]. The translocation of AIF from mitochondria to the nucleus following treatment with *V. ponticum* flower extract provides additional insights into the apoptotic cascade triggered by *V. ponticum*. AIF, a mitochondrial intermembrane protein, plays a pivotal role in caspase-independent apoptosis, where it translocates to the nucleus to induce DNA fragmentation and chromatin condensation [43,44]. Additionally, the evaluation of caspase-3 activity was conducted to determine the involvement of caspase-dependent pathways in the apoptotic effects induced by *V. ponticum* flower extract. The results indicated that caspase-3 was not activated (cleaved) following treatment with the extract, suggesting that the apoptosis observed may occur through a caspase-independent mechanism.

Assessment of ΔΨm further supports the induction of apoptosis by the extract, highlighting its therapeutic potential as a novel agent for combating NSCLC [35]. The loss of ΔΨm reflects perturbations in mitochondrial function, leading to the release of pro-apoptotic factors and initiation of apoptotic signaling cascades [35]. Notably, phytochemical studies of other species of *Verbascum*, like *V. calvum*, showed a high phenolic content, and by medium-pressure liquid chromatographic separation, both iridoid glucosides ajugol and aucubin were isolated from the methanol extract [16]. Indeed, iridoids have been extensively researched for their anti-tumor properties and therapeutic benefits in chronic conditions [45,46]. However, Aucubin has shown extensive biological effects, including antioxidant, anti-inflammatory, and anti-cancer properties. Specifically, aucubin was able to block the proliferation of A549 cells by upregulating the expression of p53 and p21 proteins to block cell cycle progression in the G0/G1 phase [47]. Furthermore, the iridoids-containing extract induced a remarkable decrease in ΔΨm leading to apoptosis of cancer cells used, such as glioblastoma [48].

Further exploration of the molecular mechanisms involved could facilitate the development of targeted therapies against NSCLC, addressing an unmet need in lung cancer treatment.

Based on this evidence, one hypothesis could be that the flower extract contains a high amount of phenolic compounds, particularly iridoids, by which it exerts its mechanism of action on tumor cells. However, phytochemical characterization is an ongoing study of *V. ponticum* to detect and isolate bioactive substances alongside further molecular-based analysis involving annexin V-FITC staining; caspase activity and employing mitochondrial pharmacology tests will be incorporated in subsequent studies with the isolated bioactive compound, to further validate the effectiveness of the *V. ponticum* cytotoxicity on NSCLC cells.

## 5. Conclusions

Overall, the findings of this study, conducted on a plant with rich traditional use in the Kurdistan region, particularly to treat burns and local inflammation, provide mechanistic insights into the anti-cancer effects of *V. ponticum* flower extract against A549 NSCLC cells. The induction of apoptosis through the activation of intrinsic apoptotic pathways was evidenced by nuclear and mitochondrial alterations, DNA fragmentation, the release of AIF, and translocation to the nucleus thus inducing tumor cell apoptosis, emphasizing the therapeutic potential of *V. ponticum* in NSCLC treatment. Further studies elucidating the specific bioactive compound composition of the extract and their molecular targets are warranted to fully exploit the therapeutic potential of *V. ponticum* in cancer therapy alongside the use of a dose-dependent relationship to better detect the range of effective concentrations of the extract. Additionally, preclinical and clinical investigations are necessary to validate the efficacy and safety of *V. ponticum*-based therapies for NSCLC.

## Data Availability

All data generated or analyzed during this study are included in the manuscript.

## Supplementary Materials

The following supporting information can be downloaded at https://www.mdpi.com/article/10.3390/life14111520/s1: Figure S1: MTS assay to investigate cytotoxic effects of *V. ponticum* flower extract on normal human bronchial epithelial cells (NL-20) and human non-small cell lung carcinoma (A549 and NCL-H460) cell lines. Figure S2: DNA fragmentation assay by agarose gel electrophoresis. Figure S3: Caspase-3 activation evaluation in A549 cell. Supplementary Material and Methods S1. Caspase-3 activation assay.
