# Supplementary material for: Verbascum ponticum (Stef.) Extract Induces Lung Cancer Apoptosis via Mitochondrial-Dependent Apoptosis Pathway
Source: Life (Basel). 2024 Nov 20;14(11):1520. doi: 10.3390/life14111520 (PMC11595628; doi:10.3390/life14111520)
Supplement: Supplementary file 1 [file life-14-01520-s001.zip › life-3144726-supplementary.pdf]

Supplementary materials

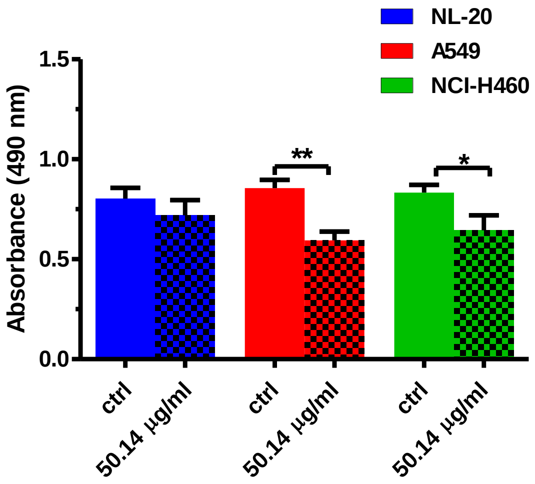

Supp. Fig. 1: MTS assay to assess cytotoxic effects of *V. ponticum* flower extract on normal human bronchial epithelial cells (NL-20) and Human non-small cell lung carcinoma (A549 and NCL-H460) cell lines. The bar graph shows both types of cells with and without incubation with 50.14 µg/ml of the flower extract for 24 hours. Data are presented as the Mean ± SD of independent quadruplicate experiments. Student *t*-test is used to compare each pair of variables. Statistical significance is indicated by $P \leq 0.05$* and $P \leq 0.01$**.

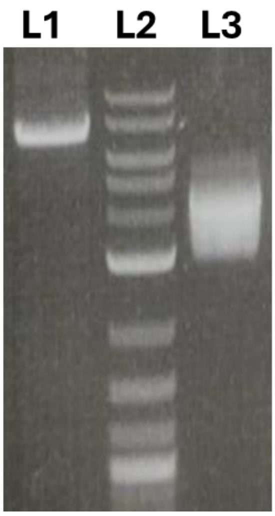

Supp. Fig. 2: DNA fragmentation assay by agarose gel electrophoresis.
(L1) untreated (control) A549 cells; (L2) DNA ladder (starting from 2000 bp); (L3) treated A549 cells with flower extract (50.14 µg/ml)-treated A549 cells for 12 hours. The image is the representative figure from three independent experiments for each condition.

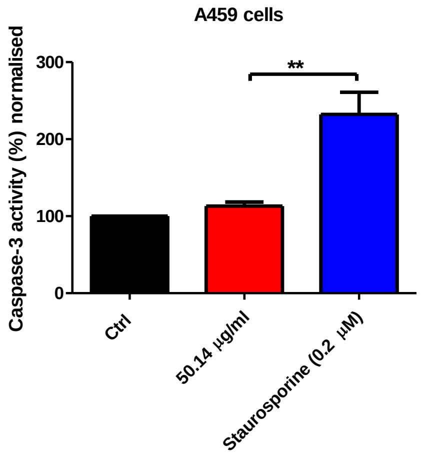

Supp. Fig. 3: Caspase-3 activation evaluation in A549 cell

Caspase-3 activity was evaluated using fluorescence microscopy after treating A549 cells with the plant extract (50.14 µg/ml). The fluorescence intensity was normalized to the control (non-treated cells), and the bar graph represents the percentage change in fluorescence intensity after 12 hours of treatment with and without the flower extract. Staurosporine (0.2 µM) was used as a positive control. Statistical significance is indicated by $P \leq 0.01$**.

Supplementary material and methods 1. Caspase-3 activation assay

A459 cells were cultured in 24-well plates on glass coverslips. Cells were fixed in 4% PFA in then incubated in 0.2% Triton X-100 (Sigma) for 5 min, after overnight incubation at 4°C with an antibody against cleaved caspase-3, the cells washed with PBS, then the cells were incubated for 1 h at room temperature with an AlexaFluor 488-conjugated goat anti-rabbit IgG antibody (Invitrogen) in 1% goat serum. Eventually, the fluorescence intensity was detected ad using a fluorescence microscope (Zeiss, Germany).
